# Screening and Functional Analysis of TEK Mutations in Chinese Children With Primary Congenital Glaucoma

**DOI:** 10.3389/fgene.2021.764509

**Published:** 2021-12-10

**Authors:** Yunsheng Qiao, Yuhong Chen, Chen Tan, Xinghuai Sun, Xueli Chen, Junyi Chen

**Affiliations:** ^1^ Department of Ophthalmology and Visual Science, Eye and ENT Hospital, Shanghai Medical College, Fudan University, Shanghai, China; ^2^ NHC Key Laboratory of Myopia, Chinese Academy of Medical Sciences, Shanghai Key Laboratory of Visual Impairment and Restoration (Fudan University), Shanghai, China; ^3^ State Key Laboratory of Medical Neurobiology and MOE Frontiers Center for Brain Science, Institutes of Brain Science, Fudan University, Shanghai, China

**Keywords:** TEK receptor tyrosine kinase, primary congenital glaucoma, next generation sequencing, mutations, functional analysis

## Abstract

**Purposes:** Recent studies have suggested that loss-of-function mutations of the tunica intima endothelial receptor tyrosine kinase (TEK) are responsible for approximately 5% of primary congenital glaucoma (PCG) cases in diverse populations. However, the causative role of TEK mutations has not been studied in Chinese PCG patients. Here, we report the mutation spectrum of TEK after screening a large cohort of PCG patients of Chinese Han origin and analyze the identified variants in functional assays.

**Methods:** TEK-targeted next-generation sequencing (NGS) was performed in 200 PCG patients. Candidate variants were prioritized by mutation type and allele frequency in public datasets. Plasmids containing wild type and identified variants of TEK were constructed and used to assess protein expression, solubility, receptor auto-phosphorylation, and response to ligand stimulation in cell-based assays.

**Results:** Ten missense and one nonsense heterozygous variants were detected by NGS in 11 families. The clinical features of TEK variants carriers were comparable to that of TEK-mutated patients identified in other populations and CYP1B1-mutated individuals from in-house database. Functional analysis confirmed four variants involving evolutionarily conserved residues to be loss-of-function, while one variant (p.R1003H) located in tyrosine kinase domain seemed to be an activating mutation. However, our results did not support the pathogenicity of the other five variants (p.H52R, p.M131I, p.M228V, p.H494Y, and p.L888P).

**Conclusion:** We provide evidence for TEK variants to be causative in Chinese PCG patients for the first time. Attention needs to be paid to TEK mutations in future genetic testing.

## Introduction

In healthy eyes, the intraocular pressure (IOP) is maintained by dynamic balance between aqueous humor production *via* ciliary body and drainage through trabecular and uveoscleral pathway (Johnson et al., 2017). Developmental defects of aqueous outflow structures can give rise to elevated IOP and early-onset glaucoma, causing buphthalmos, cornea stretching, optic disc cupping, and irreversible blindness (Lewis et al., 2017). Affecting children in the first 3 years of life, primary congenital glaucoma (PCG) is the most common form of infantile glaucoma with isolated angle anomalies (Young et al., 2020).

Genetic factors serve a critical role in the pathogenesis of PCG. To date, mutations of CYP1B1, LTBP2, MYOC, FOXC1, TEK, and ANGPT1 have been implicated in PCG patients from diverse populations (Stoilov et al., 1997; Kaur et al., 2005; Ali et al., 2009; Chakrabarti et al., 2009; Souma et al., 2016; Thomson et al., 2017). However, the molecular basis of PCG remains largely unrevealed, especially for patients of Chinese Han origin with CYP1B1 heterozygous and homozygous mutations accounting for only 17.2% of cases and no mutations of LTBP2 being identified (Chen et al., 2008; Chen et al., 2016).

Haploinsufficiency of TEK (Tunica intima endothelial receptor tyrosine kinase, Gene ID: 7010) caused by heterozygous loss-of-function mutations was reported to be causative for 5% of multiethnic PCG patients characterized by autosomal dominant inheritance with decreased penetrance and variable expressivity (Souma et al., 2016). This gene encodes a transmembrane receptor tyrosine kinase (Tie2) involved in cardiovascular development and homeostasis (Saharinen et al., 2017). Activating mutations of TEK have been identified as the genetic causes for most inherited and sporadic cases of venous malformation (VM) (Vikkula et al., 1996; Limaye et al., 2009). Notably, TEK is also expressed in the endothelia of Schlemm’s canal (SC) and collector channels, vital components of conventional aqueous outflow pathway (van Zyl et al., 2020). Conditional knockout of TEK from embryonic day 17.5 caused completely absent SC and significantly increased IOP, while heterozygous TEK deletion led to a severely hypomorphic SC and elevated IOP in mice (Souma et al., 2016). This dose–effect relationship clearly demonstrated that the normal function of Tie2 is indispensable during angle development.

For the first time, we performed TEK-targeted next generation sequencing in a large cohort of Chinese PCG patients aiming to verify the causative role and depict the mutation spectrum of TEK in this population.

## Materials and Methods

### Study Participants

Two hundred patients along with their parents were enrolled *via* the Eye and ENT Hospital Biobank from 2004 to 2018. The diagnosis of PCG must agree with the following criteria: (1) expansion of corneal diameter, corneal clouding, or Haab’s striae; (2) IOP >21 mm Hg; (3) optic nerve cupping or asymmetric cup-to-disc ratio; and (4) disease onset before age 3. Other developmental glaucoma or secondary glaucoma, such as Peter’s anomaly, Axenfeld–Rieger syndrome, and aniridia were excluded.

All PCG cases were confirmed by glaucoma specialists before surgery under general anesthesia. Anterior segment examination and corneal diameter measurement were performed with the aid of surgical microscopes. IOP was measured using a Tono-PEN tonometry (Mentor, Norwell, MA, United States). The structures of anterior chamber angle and the optic disc were observed under gonioscopy whenever possible. Five milliliters of venous blood was collected from each PCG patient and his/her parents and preserved in the biobank for genomic DNA extraction.

### Next Generation Sequencing and Variants Prioritization

In this study, a customized panel with 58 pairs of primers was designed to cover the coding regions and 50-bp sequences flanking the splice sites of TEK gene (Supplementary Table S1). DNA samples were amplified by multiplex PCR and sequenced by a Hiseq4000 platform (Illumina, San Diego, CA). Sequencing reads were aligned to the human reference genome assembly (hg19). Variants were called by Genome Analysis Toolkit (version 3.70) and annotated by ANNOVAR (Wang et al., 2010). Exonic and splice site (±5 bp) variants with an allele frequency <0.1% in the Genome Aggregation database (gnomAD_all and gnomAD_eas) were validated by Sanger sequencing and selected for further analysis. Additionally, four probands were exome sequenced using a SureSelect Human All Exon Kit (Aglient, Santa Clara, CA, United States) and the aforementioned Hiseq4000 platform. When available, parental DNA samples were also subjected to Sanger sequencing for co-segregation analysis.

### *In Silico* Functional Study

Twelve *in silico* tools were integrated to predict the pathogenicity of non-synonymous variations, including SIFT (Sim et al., 2012), Polyphen-2 (Adzhubei et al., 2010), LRT (Chun and Fay, 2009), MutationTaster (Schwarz et al., 2014), MutationAssessor (Reva et al., 2011), FATHMM (Shisha et al., 2013), PROVEN (Choi and Chan, 2015), VEST (Carter et al., 2013), Meats (Kim et al., 2017), M-CAP (Jagadeesh et al., 2016), CADD (Kircher et al., 2014), and DANN (Quang et al., 2015). Multiple Tie2 protein sequences from different species were retrieved to evaluate the evolutionary conservation of affected residues. To assess the effect of residue substitution on protein structure and stability, the FoldX plugin for YASARA (Van Durme et al., 2011) was used to calculate the free energy change induced by specific mutations with three repetitions each, and the resulting structures were visualized with YASARA View (Krieger and Vriend, 2014). For variants located within immunoglobulin (Ig)-like domains and the epidermal growth factor (EGF)-like domain, the Tie2 ligand-binding domain crystal structure (PDB ID: 2GY5, 23-445 aa) was utilized as the template (Barton et al., 2006). Additionally, crystal structure of the membrane proximal three fibronectin type III (FNIII) domains of Tie2 (PDB ID: 5UTK, 438-741 aa) and Tie2 kinase domain (PDB: 1FVR, 798-1124 aa) were used as templates for respective missense variants (Moore et al., 2017) (Shewchuk et al., 2000).

### Cloning of TEK-Expressing Plasmids

The full-length human TEK cDNA (reference sequence: NM_000459.5, 4,683 bp) with 3x Flag (DYKDDDDK) tag was cloned into the pCDNA3.1-ZsGreen vector (Supplementary Figure S1A). Site-directed mutagenesis was performed to introduce mutations into TEK-expressing vectors. Specifically, two plasmids (Y1024X-Flag and TEK1023-Flag, Supplementary Figures S1B, C) were generated to verify the nonsense mutation p.Y1024*. All plasmids were validated by Sanger sequencing.

### Western Blot Analysis

Wild-type (WT) or mutant TEK-Flag plasmids (1 μg) were transfected into subconfluent 293T cells in six-well dishes using Lipofectamine 2000 (Life Technologies, Carlsbad, CA). Twenty-four hours later, cells were lysed by 1% Triton X-100, centrifuged, and separated into supernatant and insoluble pellets. Radioimmunoprecipitation assay (RIPA) buffer was added to the pellets before sonication. Same amount of proteins (15 μg) was separated by electrophoresis and transferred to polyvinylidene fluoride (PVDF) membranes. After blocking with 5% non-fat milk, antibodies against phosphotyrosine (1:3,000; ab179530, Abcam, Cambridge, United Kingdom) and Flag (1:5,000; 20543-1-AP, Proteintech, Rosemont, IL, United States) were used to detect the phosphorylation and expression of Flag-tagged Tie2, respectively. β-Actin (1:5,000; 66009-1-Ig, Proteintech, Rosemont, IL, United States) was used as a loading control. For proteasomal inhibition assay, transfected 293T cells were treated with 5 μM MG132 (MedChemExpress, Shanghai, China) for 24 h before harvest.

### Quantitative Real-Time PCR

Total RNA was extracted from 293T cells using EZ-press RNA Purification Kit (EZBioscience, Roseville, MN, United States) 24 h after transfection. One thousand nanograms of RNA was used for reverse transcription and subsequent quantitative PCR in CFX96TM real-time system (Bio-Rad, Hercules, CA, United States). The housekeeping gene ACTB was used as the internal control. All mRNA variants were normalized to WT-TEK using the δ-delta Ct method. The primers are listed below: forward primer, 5′-TCC​AGG​CAA​CTT​GAC​TTC​GG-3; reverse primer, 5′-CCT​TGA​ACC​TTG​TAA​CGG​ATA​G-3.

### TEK Localization Assay

Human umbilical vein endothelial cells (HUVECs) were obtained from ScienCell and cultured using endothelial cell medium (ScienCell, Carlsbad, CA, United States). Cells were transfected with either WT or mutant TEK-Flag plasmids by electroporation with Neon transfection system (Thermo Fisher, MA, United States) following the manufacturer’s instructions. The transfected cells were seeded on glass coverslips in 24-well dishes. After 48 h, cells were either directly fixed with 4% paraformaldehyde for 15 min at room temperature or stimulated with 600 ng/ml recombinant human Angiopoietin 1 (R&D systems, Minneapolis, MN, United States) for another 30 min at 37°C before fixation. Cells were then permeabilized and blocked by 5% goat serum and 0.3% TritonX-100 in phosphate-buffered saline (PBS) for an hour at room temperature and incubated overnight at 4°C with anti-Flag antibody (1:200; 66008-3-Ig, Proteintech, Rosemont, IL, United States) and anti-ZO1 antibody (1:500; 21773-1-AP, Proteintech, Rosemont, IL, United States). Cyanine3- and Cyanine5-conjugated secondary antibodies (Thermo Fisher, MA, United States) were used to detect Flag and ZO-1 signals, respectively. Cell nuclei were stained by 4,6-diamidino-2-phenylindole (DAPI) (1 μg/ml; Sigma-Aldrich, St. Louis, MO, United States) for 5 min. Images were taken with a fluorescence microscope (DM4000 B, Leica, Wetzlar, Germany).

### Statistical Analysis

Statistical analysis was performed with Stata software (15.1, StataCorp). Continuous variables were compared using Student’s *t*-test or paired *t*-test, while Pearson chi-square test was applied to comparing categorical variables. A *p* value <0.05 was considered statistically significant.

## Results

### Identification of Rare/Novel TEK Variants

An average read depth of 1,938.3 was achieved in this study, while 99.3% of the targeted regions had 100x coverage per base. In total, 44 TEK variants were detected in 199 samples. Among these, 11 rare or novel heterozygous nonsynonymous variants were identified in 11 unrelated patients (Table 1 and Supplementary Figure S2). Four variants (p.R1003H, p.H52R, p.H494Y, and p.Y1024*) co-segregated with the glaucoma phenotype. Other six variants were inherited from unaffected parents, consistent with the autosomal dominant pattern with decreased penetrance and variable expressivity suggested by Souma and others (Souma et al., 2016).

**TABLE 1:** TEK variants identified in 11 families.

Family ID	Patient ID	Chromosomal position	Exons	DNA change	Amino acid change	gnomAD_all	gnomAD_eas	In silico predictions	Average ddG
01	C-9	27217702	exon19	c.3008G > A	p.R1003H	0.000012	0.000054	11|12	2.55
02	C-56	27168521	exon3	c.393G > A	p.M131I			2|12	0.81
03	C-39	27192508	exon11	c.1511T > C	p.L504P			8|12	4.16
04	C-64	27172667	exon5	c.682A > G	p.M228V	0.000032	0.000272	0|12	0.36
05	C-99	27209206	exon16	c.2663T > C	p.L888P			10|12	2.33
06	C-151	27157931	exon2	c.155A > G	p.H52R	0.000028	0	0|12	−0.55
07	C-165	27172715	exon5	c.730C > T	p.P244S			8|12	4.00
08	C-270	27206737	exon15	c.2522C > T	p.A841V	0.000004	0	11|12	1.40
09	C-290	27190679	exon10	c.1480C > T	p.H494Y			0|12	−0.55
10	C-350	27173250	exon6	c.791G > T	p.C264F			10|12	8.48
11	C-354	27218784	exon20	c.3072T > A	p.Y1024X			3|3	NA

Chromosomal positions correspond to GRCh37/hg19 assembly. Reference TEK mRNA, sequence, NM_000459.4. Reference TEK, protein sequence, NP_000450.2. gnomAD_all, allele frequency in all populations from gnomAD, dataset. gnomAD_eas, allele frequency in East Asian populations from gnomAD, dataset. *In silico* predicitons were made combining 12 programs listed in *Methods*, and presented as “Number of deleterious predictions | Number of total predictions”. Changes in Gibbs free energy were calculated via FoldX plugin for YASARA (average of three repetitions).

One mutation (p.A841V) was previously identified in a large American pedigree by Young et al. (2020). In addition, a different amino acid change in proline residue 244 (p.P244R) was also reported in the same study. The nonsense mutation (p.Y1024*) was confirmed by Western blotting (Supplementary Figure S1D). The truncated protein lacks tyrosine residues for phosphorylation in C-terminus (e.g., 1,102 and 1,108), which are indispensable in mediating cellular signals. Therefore, it was reasonable to speculate that these variants were detrimental to protein function.

The remaining eight missense variants differed in pathogenicity prediction. Half of them (p.C264F, p.L504P, p.L888P, and p.R1003H) involved evolutionarily conserved residues (Supplementary Figure S3) and evaluated as destabilizing by FoldX (Table 1), whereas the other four variants (p.H52R, p.M131I, p.M228V, and p.H494Y) were predicted to be benign by most *in silico* tools.

Since family 01-07 were previously confirmed negative for CYP1B1 and LTBP2 mutations, probands from family 08-11 were exome sequenced to explore mutations in other known disease-causing genes. C-354 was found to inherit the nonsense mutation of TEK (p.Y1024*) and a missense variant of CYP1B1 (p.L107V) from his affected father. C-350 carried an additional missense variant of LTBP2 (p.T886K) from her mother compared to her unaffected father. No rare or novel variants of MYOC, FOXC1, and ANGPT1 were detected (Supplementary Table S2).

### Clinical Characteristics of TEK-Mutated PCG Patients

In this study, 5.5% of PCG patients carried rare or novel variants of TEK (Table 2). Of the carriers, 13 out of 19 (68.4%) exhibited clinically identifiable glaucoma phenotypes starting from 9.5 months after birth on average. There appeared to be a gender predilection, as most cases were male. In addition, more patients were bilaterally affected. Eight patients were followed up for disease prognosis (Table 3). Most of them (5/8) remained stable after initial trabeculotomy. Two patients underwent secondary angle surgeries, and one was enucleated for ocular components.

**TABLE 2:** Comparison of clinical characteristics of TEK-mutated PCG cohorts.

	Souma et al. (2016)	Young et al. (2020)	This study	*p*-value
Mutation rate (%)	10/189 (5.3)	NA	11/200 (5.5)	0.931^a^
Penetrance (%)	14/22 (63.6)	13/21 (61.9)	13/19 (68.4)	0.979^a^
Sex (male: female)	9:5	6:11	8:3	0.103^a^
Bilaterally: unilaterally involvement	6:6	10:4	7:4	0.470^a^
Age at onset (month, mean ± SD)	3.73 ± 7.1	7.27 ± 7.2	9.55 ± 10.2	0.162^b^

a Pearson’s chi squared test.

b Kruskal–Wallis test.

**TABLE 3:** Clinical information for TEK-mutated patients.

Family ID	Patient ID	Amino acid change	Sex	Age at onset	Eyes	Preoperative IOP (mmHg)	Cornea clouding	Corneal diameter (mm)	Cup–disc ratio	Treatment
01	C-9	p.R1003H	M	Birth	OD	20.6	No	12	0.5	Loss to follow-up
					OS	20.6	Haab’s striae	13	0.7	
02	C-56	p.M131I	M	2y	OD	20.6	Macula	13	0.4	1 trabeculotomy
03	C-39	p.L504P	M	2y	OD	31	Clear	14.5	0.4	1 trabecolotomy in both eyes
					OS	48.5	Yes	16.5	0.9	
04	C-64	p.M228V	F	3 m	OD	34	Yes	14.5	0.9	Initial trabeculotomy and loss to follow-up
					OS	25	Yes	14.5	0.8	
05	C-99	p.L888P	M	2y	OD	50.62	Haab’s striae	14	0.8	2 trabeculotomies in both eyes
					OS	54.66	Haab’s striae	13	0.8	
06	C-151	p.H52R	F	Birth	OD	–	–	–	–	1 trabeculotomy and 1 trabecolectomy in both eyes
					OS	–	–	–	–	With subsequent tube shunt and enucleation of the left eye
07	C-165	p.P244S	M	Birth	OS	28	–	13.5	1.0	1 trabeculotomy
08	C-270	p.A841V	M	2 m	OS	42	Yes	13	1.0	1 trabeculotomy
09	C-290	p.H494Y	M	1y	OS	45	Yes	13	1.0	2 trabeculotomies
10	C-350	p.C264F	F	1y	OD	28	Haab’s striae	13.5	1.0	1 trabeculotomy in both eyes
					OS	30.4	Haab’s striae	13.5	1.0	
11	C-354	p.Y1024X	M	4 m	OD	31.8	–	13	–	Initial trabeculotomy in both eyes and loss to follow-up
					OS	29	–	12.5	–	

Sex: M, male; F, female. Age at onset: m, month; y, year. Eyes: OD, right eye; OS, left eye.

There were no statistically significant difference between TEK-mutated PCG groups with regard to clinical features listed in Table 2. Similarly, the clinical presentations were comparable between Chinese patients with TEK mutations and those with CYP1B1 mutations or unidentified causes (Table 4).

**TABLE 4:** Comparison of clinical characteristics of PCG subgroups.

	CYP1B1	TEK	Unknown causes	*p*-value
Mutation rate (%)	20/124 (16.1)	11/200 (5.5)	NA	0.004^a^
Sex (Male: Female)	14:6	8:3	68:29	0.983^a^
Bilaterally: unilaterally involvement	12:8	7:4	46:51	0.398^a^
Age at onset (month, mean ± SD)	11.18 ± 13.04	9.55 ± 10.2	11.84 ± 12.49	0.647^b^
Preoperative IOP (mmHg)	40.94 ± 11.03	35.34 ± 5.89	35.45 ± 11.73	0.143^b^
Corneal diameter (mm)	13.26 ± 1.30	13.78 ± 1.15	13.35 ± 1.00	0.587^b^
Age at operation (day)	972.21 ± 1008.5	665.3 ± 564.5	718.51 ± 703.3	0.876^b^

a Pearson’s chi squared test.

b Kruskal–Wallis test.

### Functional Impact of TEK Variants

**FIGURE 1:**
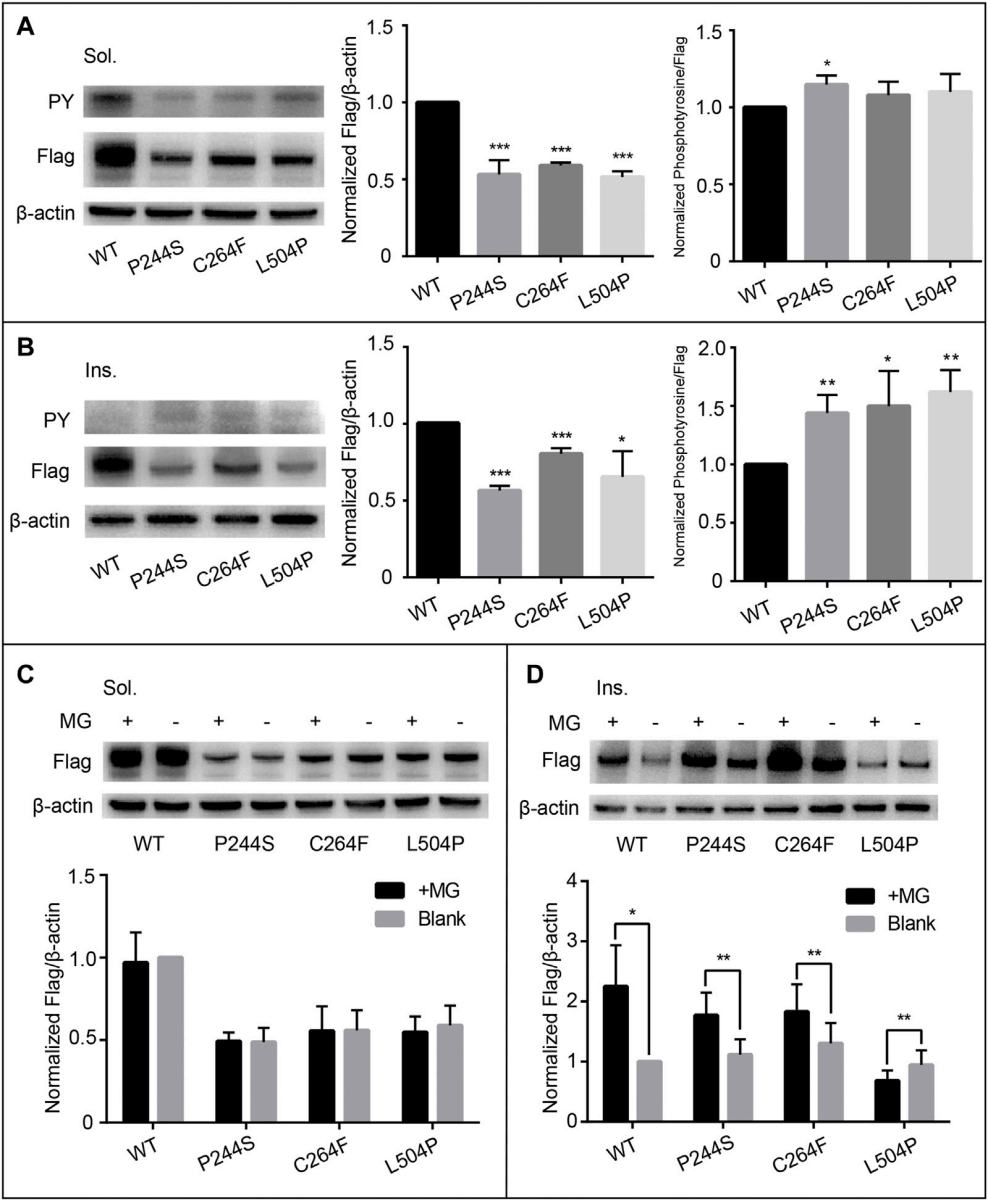
Functional assessment of variants p.P244S, p.C264F, and p.L504P. The expression of soluble protein was significantly reduced **(A)** and remained unchanged after MG132 treatment **(C)**. No dramatic change in auto-phosphorylation was detected **(A)**. The expression of insoluble protein was also significantly reduced with increased phosphorylation level **(B)**. In addition, protein degradation was reversed in p.P244S and p.C264F, while p.L504P was unresponsive to proteasomal inhibition **(D)**. Sol, soluble; Ins, insoluble; PY, phosphotyrosine; WT, wild type; MG, MG132.^a

**FIGURE 2:**
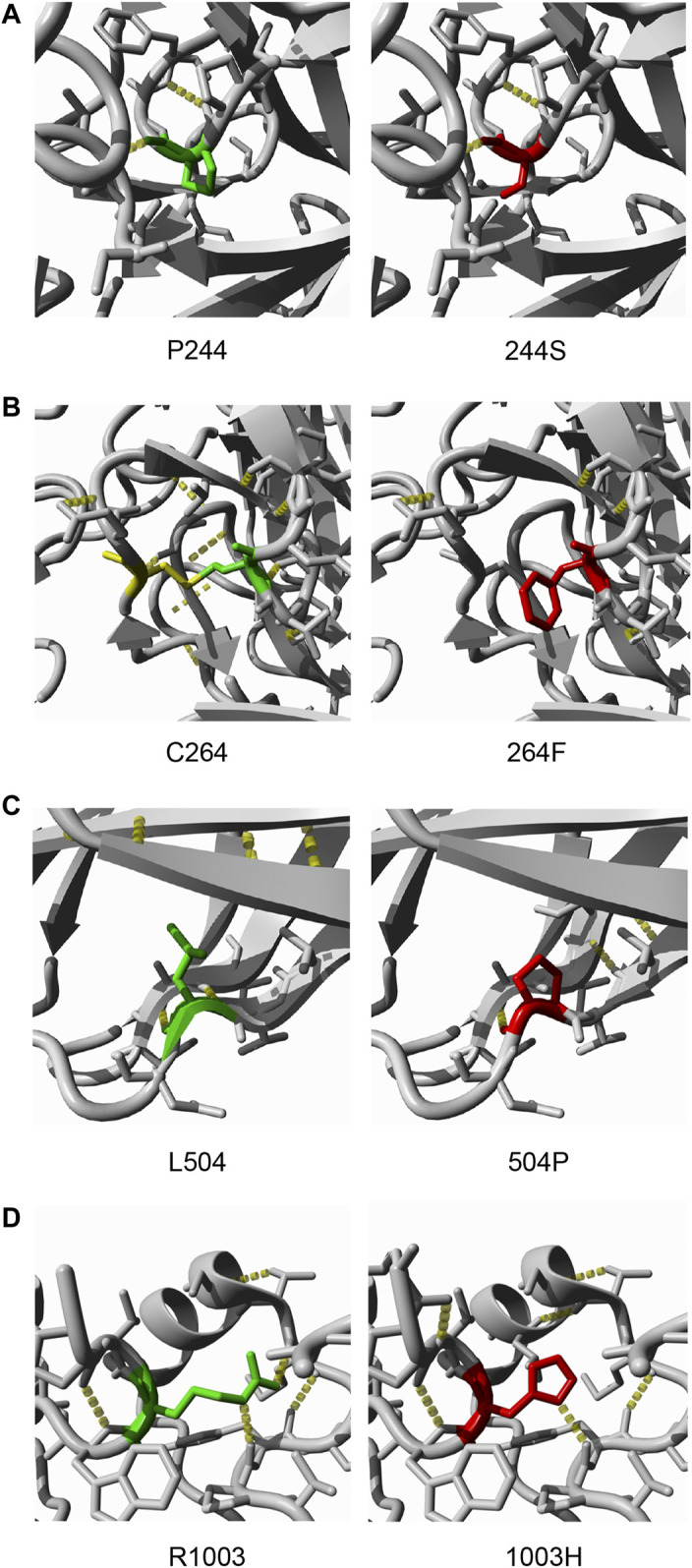
Crystal structures of four pathogenic TEK mutants **(A)**. p.P244S substitutes a nonpolar proline to polar serine, which may influence the structure of a β-turn **(B)**. Cysteine-264 forms a disulfide bond with Cysteine-255. Thus, substitution of C264 was predicted to be destabilizing due to the disruption of a disulfide bond **(C)**. p.L504P is within a β-sheet of the FNIII domain; the substitution is likely to cause loss of hydrogen bonds, as is the case in p.R1003H **(D)**. Green, wild-type residue; red, mutated residue; yellow, cysteine-255; yellow dots, hydrogen bonds.

All PCG-related TEK missense variants but p.A841V were tested in cell-based assays to evaluate their impact on protein expression, solubility, auto-phosphorylation, and response to ligand stimulation. First, WT and mutant TEK plasmids were overexpressed in 293T cells. The transcription levels of missense variants were all comparable to WT (Supplementary Figure S4). Three variants (p.P244S, p.C264F, and p.L504P) located in the ectodomain of Tie2 led to a dramatic reduction in protein expression in both soluble and insoluble fractions (Figure 1). After inhibition of proteosomal activity by MG132, the insoluble fraction of p.P244S and p.C264F mutant was remarkably increased, suggesting significant protein degradation, whereas p.L504P was insensitive to MG132 treatment. P244 together with P243 is critical in the structure of a β-turn (Figure 2). Substitution of proline (non-polar) to a serine (polar) is likely to change protein conformation. As C264 forms a disulfide bond with C255, we inferred that the p.C264F variant might lead to the disruption of the disulfide bridge and misfolding of translation product. L504 locates in the E β-strand of fibronectin type IIIa (FNIIIa) domain, which contributes to a classical antiparallel β-sheet motif (Moore et al., 2017). The replacement of leucine by proline disrupted intra-strand hydrogen bond resulting in an energetically unstable structure.

**FIGURE 3:**
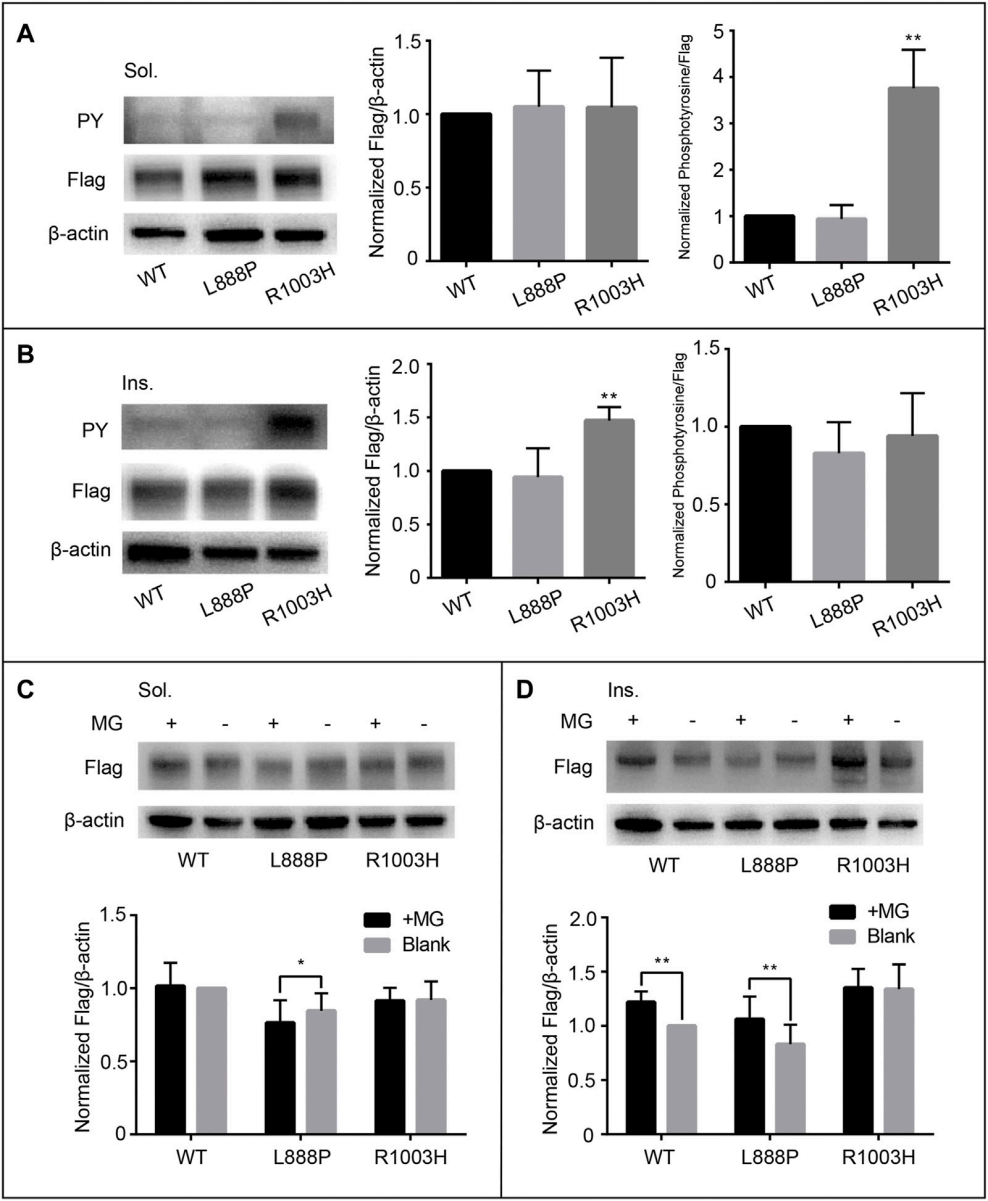
Functional assessment of cytodomain variants p.L888P and p.R1003H. The protein expression and auto-phosphorylation levels of p.L888P are comparable to that of wild type **(A**, **B)**. However, p.R1003H is significantly phosphorylated in soluble portion **(A)** and enriched in insoluble fraction **(B)**, which does not respond to MG132 treatment **(C, D)**. Sol, soluble; Ins, insoluble; PY, phosphotyrosine; WT, wild type; MG, MG132.

Both protein expression and auto-phosphorylation levels of the other four variants in the ectodomain (p.H52R, p.M131I, p.M228V, and p.H494Y) were comparable to that of WT (Supplementary Figure S5), consistent with the *in silico* prediction of pathogenicity and structural analysis (Supplementary Figure S6). Notably, p.R1003H mutant was overexpressed in the insoluble component with increased auto-phosphorylation in the soluble fraction, matching the features of activating mutations (Figure 3). Structural analysis indicated that the *de novo* variant p.R1003H was likely to cause the disruption a hydrogen bond connecting R1003 with M1024 (Figure 2). However, variant p.L888P could not be distinguished from WT in our cell-based assay (Figure 3).

**FIGURE 4:**
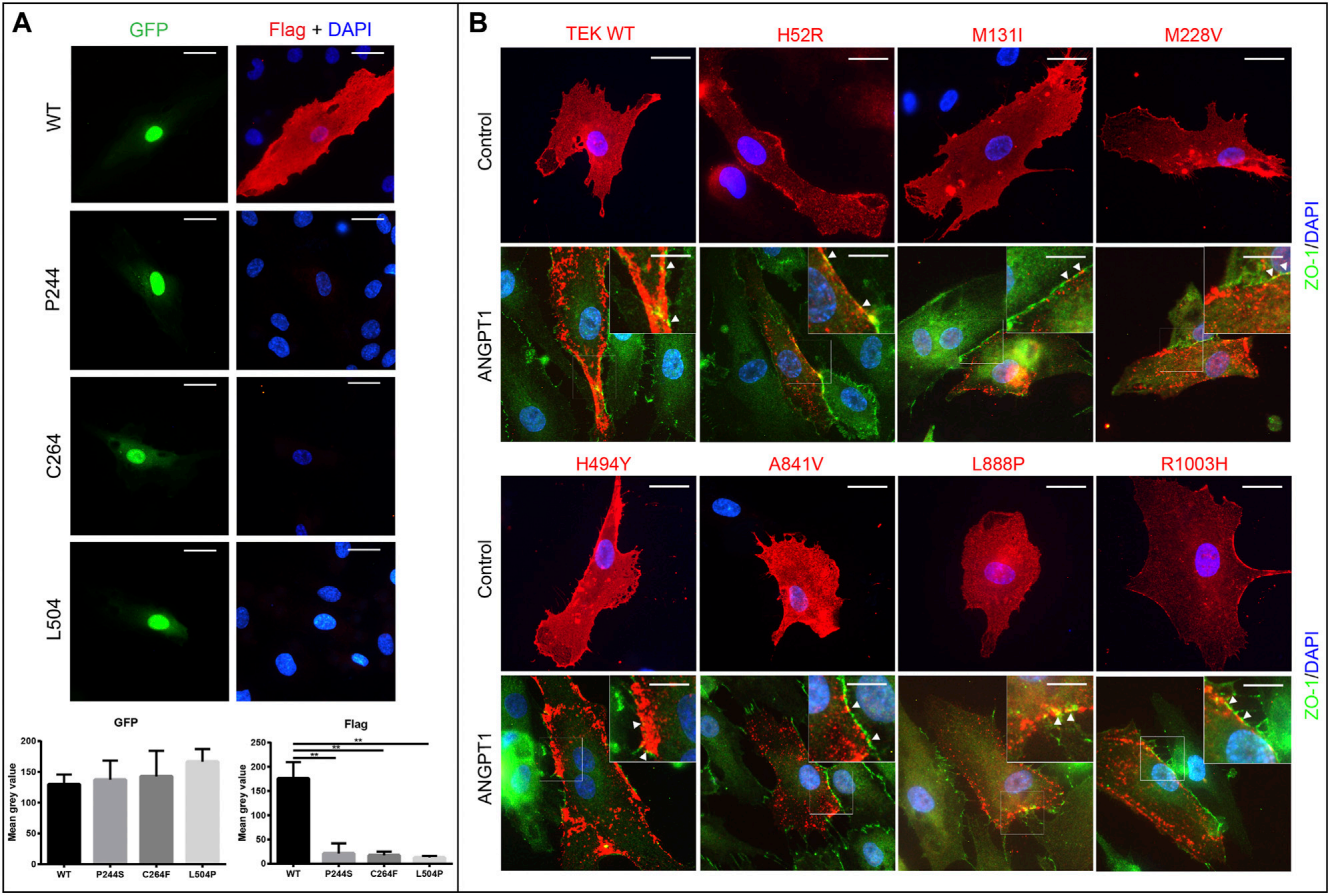
Expression and ligand responsiveness of TEK variants **(A)**. Expression of p.P244S, p.C264F, and p.L504P were greatly decreased in HUVECs. GFP (green) signals indicated cells were successfully transfected. Wild-type Tie2 (anti-Flag, red) was diffusely distributed in cellular membrane of quiescent HUVECs (first row). However, Flag signals for three loss-of-function variants were notably reduced. Scale bars indicate 25 μm. **(B)** After stimulation by Angiopoietin-1 for 30 min **(lower panel)**, TEK proteins were relocated to cell–cell junctions as indicated by anti-ZO-1 (green). Nuclei were stained by DAPI (blue). Scale bars indicate 25 μm in upper panel and 12.5 μm in lower panel for magnified images. Pictures presented were representative of four biological replicates.

The ectodomain of Tie-2 consists of three Ig-like domains and an EGF-like domain, which are responsible for ligand binding (Yu et al., 2013), and a FNIII domain that is involved in ligand-induced receptor multimerization (Moore et al., 2017). Variants in these regions have the potential to weaken or disrupt ligand-mediated signaling. Thus, we transiently expressed all variants in HUVECs and explored their response to stimulation by potent agonist angiopoeitin-1 (ANGPT1). As anticipated, the WT TEK was diffusely expressed on the cytomembrane of HUVECs and relocalized to cell–cell junctions when treated with ANGPT1 (Figure 4). Surprisingly, the expression of variants p.P244S, p.C264F, and p.L504P was greatly reduced, consistent with the results of Western blotting. However, the other seven mutants did not exhibit significant alteration in response to ligand stimulation (Figure 4). A summary of functional analysis results is provided in Supplementary Table S3.

## Discussion

In this study, we screened for TEK mutations in a large cohort of Chinese PCG patients for the first time. Deep-targeted NGS yielded 10 rare/novel potentially pathogenic variants and one previously confirmed mutation. Subsequent cell-based functional study was able to classify four variants (p.P244S, p.C264F, p.L504P, and p.Y1024*) as loss-of-function, one (p.R1003H) as gain-of-function, while the remaining 5 (p.H52R, p.M131I, p.M228V, p.H494Y, and p.L888P) appeared to be benign.

The development of the vascular and lymphatic system relies upon the orderly expression of TEK (Dumont et al., 1994; Thomson et al., 2014). As an essential part of aqueous humor drainage pathway, SC shares striking similarities with lymphatic vascular system (Aspelund et al., 2014). Indeed, some studies suggested SC as a specialized hybrid vessel with both lymphatic and vascular features (Ramos et al., 2007; Kizhatil et al., 2014). However, it was not until 2014 that an unintentional discovery linked the unique properties of SC and the pathogenesis of PCG (Thomson et al., 2014). Further studies delineated a clear dose effect between TEK function and SC morphology (Souma et al., 2016). Since then, more loss-of-function TEK mutations were reported in PCG patients with different ethnic backgrounds (Kabra et al., 2017; Young et al., 2020).

Despite being exhaustively investigated, the genetic cause of PCG remained largely unknown for most outbred populations (Mashima et al., 2001; Lim et al., 2013; Mohanty et al., 2013). In China, mutations in confirmed pathogenic genes only accounts for approximately 20% of PCG patients (Chen et al., 2008; Chen et al., 2016). Our results depicted the mutation spectrum of the recently reported causative gene and suggested the necessity of incorporating TEK in targeted panel for genetic diagnosis of PCG.

The incidence of TEK mutations in this study was similar to that of earlier reports (Souma et al., 2016; Kabra et al., 2017; Young et al., 2020). In addition, identified variants were predominantly located in the ectodomain of Tie2 (7/11), a distinctive feature varying from those associated with familial or sporadic VM (Saharinen et al., 2017). Possibly due to the wide coverage of coding sequence (23 exons, 4683bp for longest transcript), disease-associated variants rarely recur. Nevertheless, one of our patients shared a missense mutation (p.A841V) with a multigeneration pedigree reported by Young et al. (2020). This family was unique in terms of high disease penetrance, complex phenotypes, and refractoriness to treatment. A modifier gene, SVEP1, was proposed to explain the remarkable clinical features. Carrying the same TEK mutation, proband from family 8 presented relatively mild symptoms, such as unilateral involvement, limited penetrance, and favorable prognosis. Subsequent WES failed to detect any variants in SVEP1, which possibly explained the phenotypic disparities.

Based on the conservation analysis, missense variants identified in this study can be categorized into two groups. Variants p.H52R, p.M131I, p.M228V, and p.H494Y involve revolutionarily un-conservative residues, while the rest locates in conserved positions. p.H52R and p.H494Y both co-segregated with phenotype; however, *in silico* predictions and functional studies did not support their causative roles. Previous structural analysis revealed that the ligand–receptor interface is confined to the top of the Tie2 Ig2 domain, including the loops B-C (147-150 aa), C, (158–161 aa), and F–G (195–200 aa), strand C (151–157 aa), and strand C(162–167 aa) (Yu et al., 2013). Thus, amino acid changes in position 52, 131, and 228 are theoretically function sparing. Of note, we identified a rare *de novo* activating mutation (p.R1003H) in one patient. Unfortunately, we could not confirm whether this patient presented with VM or not since she was lost to follow-up. However, based on previous reports, this variant should not be considered as the cause of PCG. Similarly, there was not enough evidence to classify variant p.L888P to be pathogenic judging from structural and functional analysis. There might be other mechanisms through which those variants could lead to PCG. For instance, variants in other genes involved in ANG-Tie2 pathway could contribute jointly to the phenotype. It is necessary to screen relevant genes in future studies. Another possibility is that those variants could interfere the expression of TEK gene *in vivo*, which might be revealed using gene-edited animals. Additionally, TEK variants are likely to influence the phosphorylation level of particular amino acid sites (Souma et al., 2016). To this end, technologies of proteomics are required (e.g., mass spectrometry).

Mutant TEK proteins were previously shown to exhibit diminished or reduced interaction with CYP1B1, suggesting a potential digenic inheritance (Kabra et al., 2017). Interestingly, coexistence of variants in different pathogenic genes for PCG was noticed in our patients. The CYP1B1 variants that co-occurred with TEK mutations in this study were also found in healthy controls from Asian populations (Kim et al., 2011; Gong et al., 2015; Do et al., 2016). Nevertheless, how the cytoplasmic receptor Tie2 comes into direct contact with endoplasmic reticulum-targeted enzyme and what is the biological implication of this protein complex require further investigation.

There are some shortcomings to our study. First, limited by sequencing methods, it is outside the scope of this study to illustrate the mutation profile of other known disease-causing genes or uncover new potentially causal genes in this cohort. Considering the critical role of ANG-Tie2 signaling in SC development, it is tempting to screen genes encoding other component involved in this pathway (e.g., ANGPT1, ANGPT2, ANGPT4, and TIE1). In addition, the number of patients identified with TEK mutations was small, which limited the statistic power of comparison between patient groups and precluded any meaningful genotype–phenotype association analysis.

In summary, we confirmed the causative role of TEK mutation in Chinese PCG patients for the first time. The majority of disease-associated mutations are heterozygous missense variants involving evolutionarily conserved amino acid residues. Although TEK mutations account for <5% of total cases, due attention should be given in future genetic testing.

## Data Availability

The original contributions presented in the study are publicly available in NCBI under accession number PRJNA762473.
