# Supplementary material for: Screening and Functional Analysis of TEK Mutations in Chinese Children With Primary Congenital Glaucoma
Source: Front Genet. 2021 Dec 10;12:764509. doi: 10.3389/fgene.2021.764509 (PMC8703195; doi:10.3389/fgene.2021.764509)
Supplement: Supplementary file 1 [file DataSheet1.docx]

Supplementary Material

# Supplementary Figures


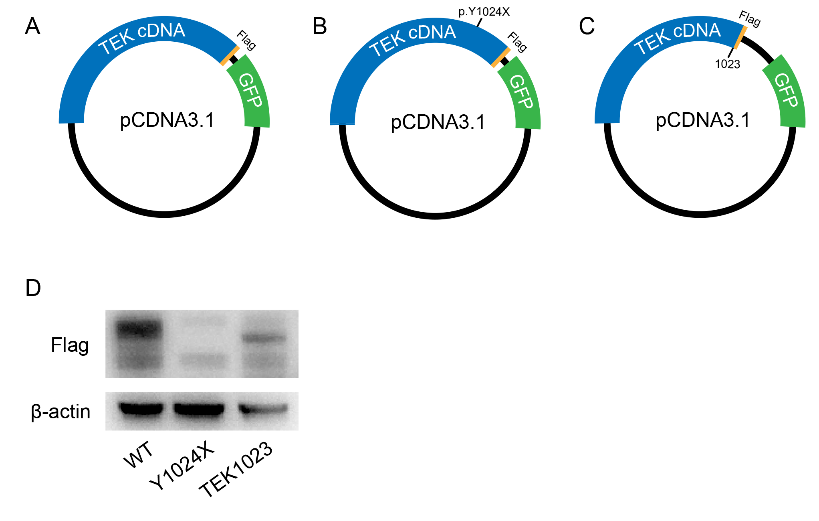


**Supplementary Figure 1.** Schematic illustrations of TEK plasmids structures. (A) Wild type and missense variants of TEK cDNA were cloned into pCDNA3.1 vector with 3xFlag tag. Green fluorescent protein (GFP) was used as an indicator for successful transfection and expression of exogenous genes. Especially, two kinds of plasmids (B) and (C) were constructed to confirm the nonsense mutation p.Y1024*. The premature stop codon was introduced into plasmid (B) which was termed as Y1024X. The TEK cDNA was truncated artificially followed by Flag and GFP sequences in plasmid (C) which was termed as TEK1023. As shown by western blotting (D), no Flag signal could be detected for Y1024X while TEK1023 was reduced in molecular weight.


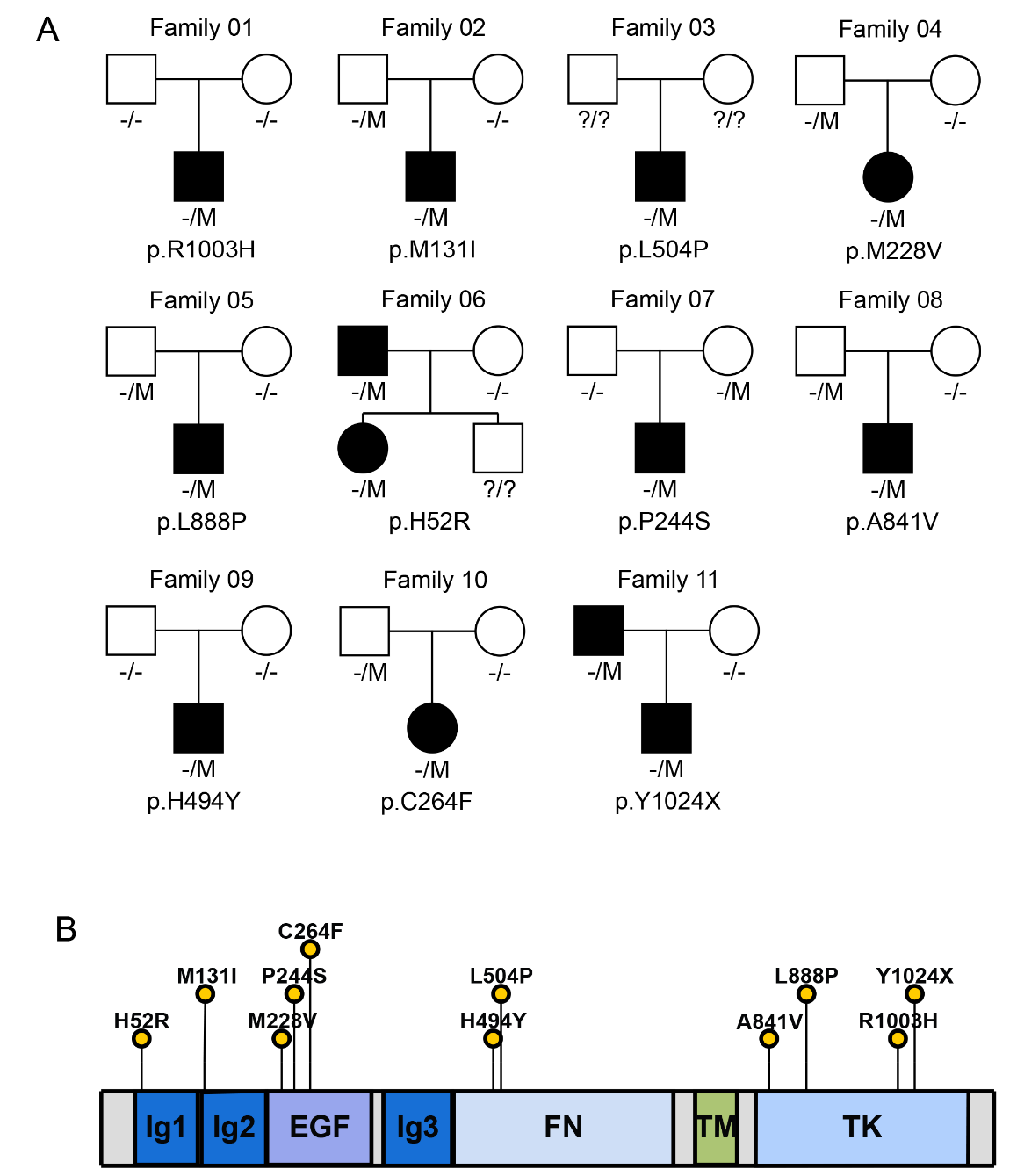


**Supplementary Figure 2.** TEK mutations identified in 11 families. (A) Pedigrees of 11 families with TEK rare/novel variants. Affected individuals are indicated by solid black squares (male) or circles (female). “-” and “M” refers to wild type and mutated alleles, respectively. Unknown genotypes are marked as “?/?”. (B) A schematic illustration of the Tie2 protein with the distribution of identified variants. Tie2 contains three immunoglobulin (Ig)-like domains, an epidermal growth factor (EGF)-like domain, a fibronectin type-III (FNIII) domain, a single-pass transmembrane (TM) domain which followed by an interrupted tyrosine kinase (TK) domain at the C-terminus.


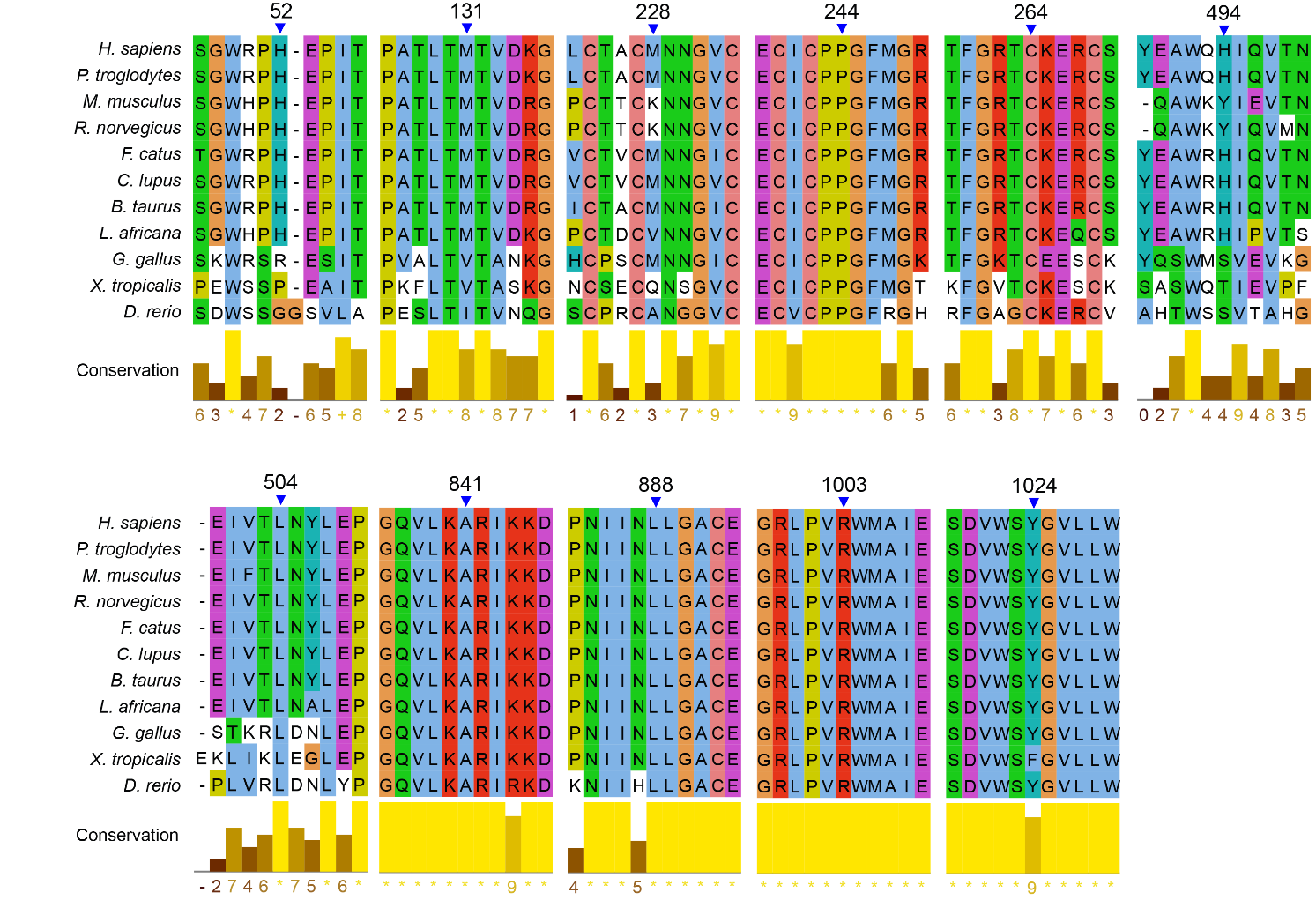


**Supplementary Figure 3.** Conservation of orthologous protein sequences at TEK variants locations. Multiple protein sequence alignment across 11 species are shown for 11 TEK variants identified in this study. Histograms below show the degree of conservation at each residue. Proline 244, Cysteine 264, Leucine 504, Alanine 841, Leucine 888, and Arginine 1003 were strictly conserved.


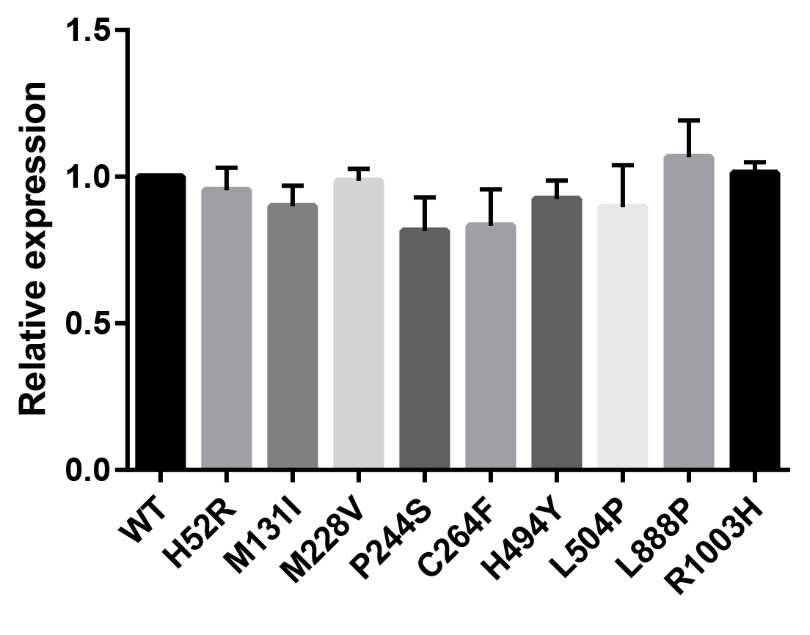


**Supplementary Figure 4.** Transcription levels of 9 missense variants. The concentration of mRNA for 9 missense variants are comparable to that of wild type. Disparities of protein expression are not caused by difference in transfection efficiency.


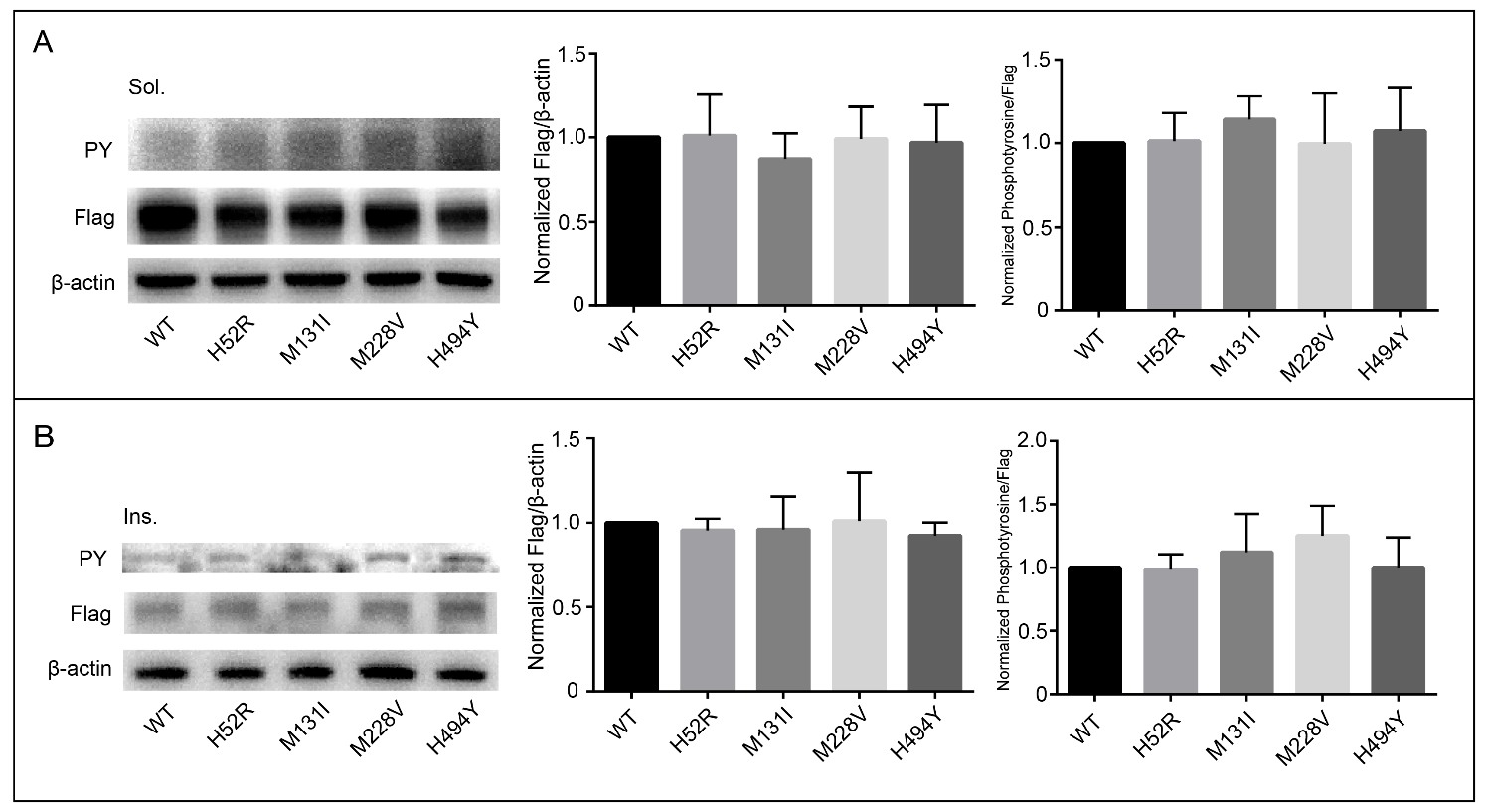


**Supplementary Figure 5.** Functional assessment of variants p.H52R, p.M131I, p.M228V, and p.H494Y. The protein expression and auto-phosphorylation levels of these 4 variants are indistinguishable to that of wild type in both soluble (A) and insoluble (B) fractions. Sol., soluble; Ins., insoluble; PY, phosphotyrosine; WT, wild type.


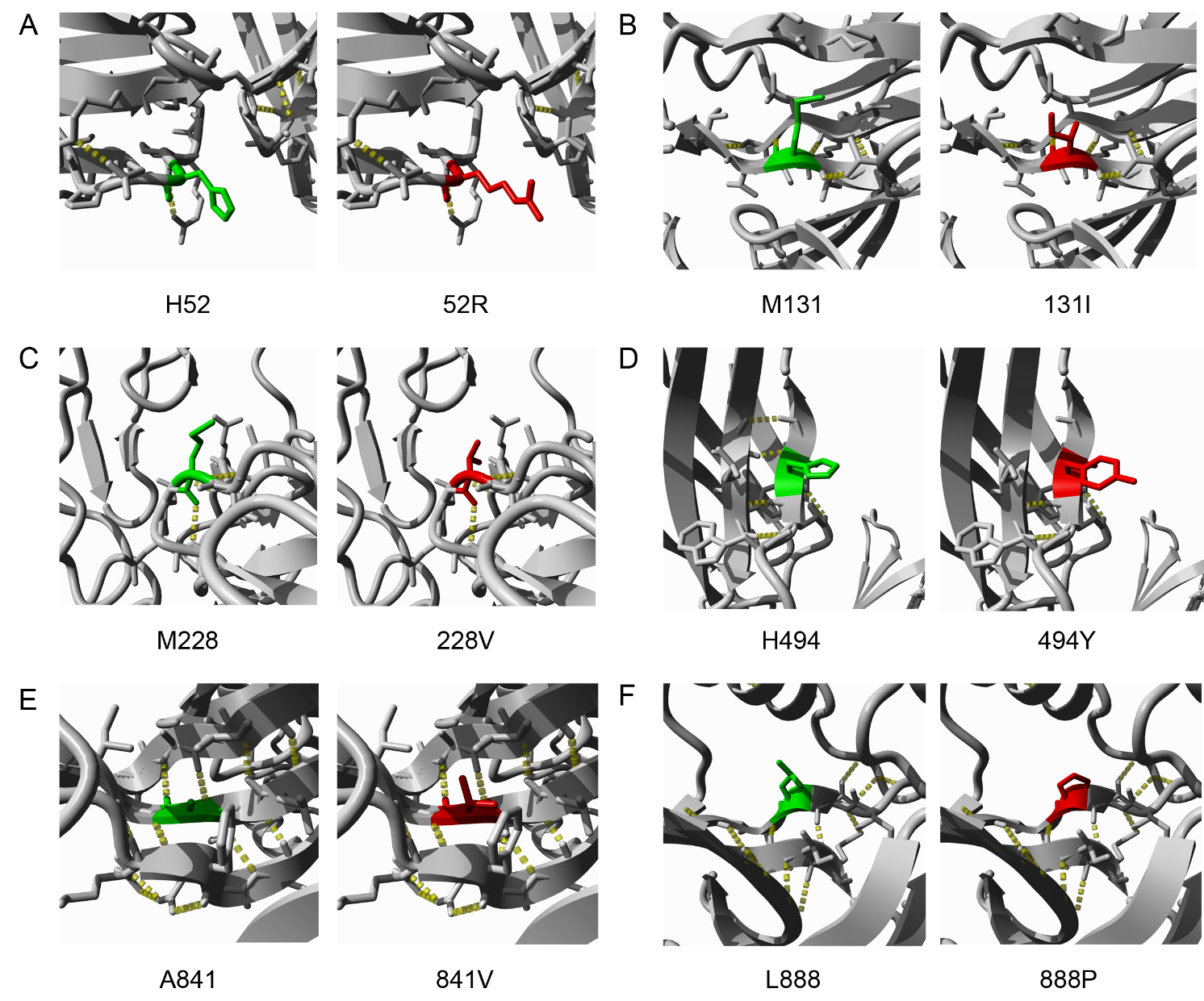


**Supplementary Figure 6.** Crystal structures of 6 missense TEK mutants. Structural analysis does not reveal apparent impact of protein stability of variants p.H52R (A), p.M131I (B), p.M228V (C), p.H494Y (D), p.A841V (E), and p.L888P(F). Green, wild-type residue; Red, mutated residue; Yellow dots, hydrogen bonds.

# Supplementary Tables

**Supplementary Table 1**. PCR primers for target region capture and amplification.

| Number | Forward Primer Sequence | Reverse Primer Sequence | Length |
| --- | --- | --- | --- |
| 1 | GTCAGAGATCCCAGTAGCCC | CCATCTCGCAATTCATCAAAACACTT | 249 |
| 2 | CCGGGACCCACACTTCCAAC | GGCTGTAACAGTCTCATTTCTGTCTGA | 243 |
| 3 | ACAGCCTTTCCCATCCTAATCTACAAA | CCCCAAATCTCTCCATCCAGT | 234 |
| 4 | ACCGCTGGGTTTTTGAAAGGA | ACCCCTTGTTCATCAGCACT | 229 |
| 5 | ATAGGCACATGGTCAGAATAGCATT | CGCCACCCAGAGGCAAT | 232 |
| 6 | CCCTACCTCTTGTATCTGATGCTG | CTCTCCTCGAACTCGCCCTT | 235 |
| 7 | AAAAGGCTAGTAAGATCAATGGTG | AGGCAAGATCGTCAAGCTCT | 218 |
| 8 | CTGACACCTAGCAAGTGCC | TACTGGGGAAAGCAATGAAACACA | 249 |
| 9 | AAGAAGATGCAGTGATTTACAAA | ACATCTGCCCACAAGACCAC | 126 |
| 10 | TGAGAGCACATTTTCTTGACCAT | CCTCCTATATACCTGGCCGAG | 225 |
| 11 | AGTACACCTGCCTCATGCTC | ACAACCTCCAACCAATGCCT | 227 |
| 12 | CTGACACACAGTAGGTGCTC | ACACGTCCTTCCCATAAACCC | 205 |
| 13 | CGAATGCGCTCTACTCACC | CTGACCTAACAGAGGCCATT | 292 |
| 14 | CTGCCATGAAGATACTGGAGAATGC | ACCTAACAGAGGCCATTTAGCG | 178 |
| 15 | ATGTTCATCCTACCATGCCACA | TGGCACAGGAACACCCAT | 248 |
| 16 | CAGAACTTGTAAAGAAAGGTGCAG | AGCTAAGCAGTCCAGGTATATCCGA | 223 |
| 17 | CCCCTACCTTACACAAATCAGC | GAGCCCCTGCCATCCTG | 236 |
| 18 | TTACGGGCCAGATTGTAAGC | ATTCCCAACTTCTGGATGGA | 240 |
| 19 | TTCTTGCCCGGTGCAT | ACTGTCCCATCCGGCTTC | 247 |
| 20 | TGGATTTGCCAGATCATATAGAAGT | AGCCAACAACACACTAGCCTA | 245 |
| 21 | TCAATGTTATGGACCTTTGCG | GGGCTTTTCCACCATCCCA | 194 |
| 22 | CATTTCTCAGTAGCCATATTCACCA | AGGCTTACTATCCTGGAAATTACCC | 246 |
| 23 | AACTTTAAGAGGACTTTGTTGGAC | AGTGATTAACGGGTTTGTATAGAAGC | 249 |
| 24 | ACACTGGACATAACTTTGCTGTCA | CTGCAACAGCCACCACCTTGAG | 248 |
| 25 | ATGGAATTGCCTCTCTGTTTCACTAAGAC | TCACAGGTCCAGGATGCCCTT | 246 |
| 26 | AATATGAACTCTGTGTGCAACTGGTCCG | CCCACCCATTCAAAAGCGAACTCC | 234 |
| 27 | GCCCCACCTCCAACACT | CAACATAAAAGTCATCTTCCGAGCTTG | 215 |
| 28 | CCTGCCTAAAAGTCAGACCAC | GTTGACTCTAGCTCGGACCAC | 201 |
| 29 | AGTTCCAGGCAACTTGACTTCG | CCAATAGTCCTGCAGTGATGT | 221 |
| 30 | TTGCCTTATATGAGCTGACATGAACT | TGCCTTGAACCTTGTAACGGAT | 224 |
| 31 | CCTCAACCAGAAAACATCAAGATTTCC | GGTTGCTTGACCCTATGTTGTTC | 249 |
| 32 | CCTAGAGCCTGAAACAGCATACCAG | ATGCCTGTCCATTCAACCAC | 227 |
| 33 | AGGCTGCTGTTAAGTTCCCA | GGGCCATTCTCCTTTGCACA | 244 |
| 34 | ATCCTTGGCTCTGCTGGAATGACC | AGCCAAAGAGAAGATGAGGCTTG | 249 |
| 35 | ATTTCTTTTTTCATCTGGTGTGGATGC | ATTGCCCTCCCCAATCACAT | 238 |
| 36 | ACCCAGATCCTACAATTTATCCAGT | ACCATGCCAATTTTTTGTAAGACCA | 249 |
| 37 | TTGTATACAGTTGATGGTGACT | ACAGGAAAAGAGCATCTTACCTCG | 246 |
| 38 | AGTTCTTTGTAAACTTGGACACCATC | AATGGGACTAGACAGACATTCAGA | 212 |
| 39 | TCCTGTCCCCCAGTGCTCCC | CGTCTCCAGCACACGGCTCT | 226 |
| 40 | CCCATGGAAACCTTCTGGACT | AGGTTGACAGACCCTACAAACA | 248 |
| 41 | ACTTTAAGGGAACTGGATTGTGAC | TACACCTCTTGACCTCGGGAC | 228 |
| 42 | TCATTAGGCTGAAAATACATAATGTTT | AGTCACCACAGAGCACTGACA | 248 |
| 43 | CACATGTAGCTGGGACATACACAA | CCTTGGCAATAAGAAGTTTACTCACAC | 234 |
| 44 | ACCCTGTCCCAGTTAAGTGA | TTTTAGTTACCTTTGTTCCCGAGAGC | 232 |
| 45 | CTGTGTGCAAGGGCCTATCCTA | GCTAGAGGCTTCCACTCACT | 244 |
| 46 | ATGCTCACCCTCTCTTGCCAT | CAGGGGCTTCTCCAGTCTGT | 200 |
| 47 | TGTCTTCCAGGAGGCACACC | AGTGTATACCGGCTGACTTTGCTA | 201 |
| 48 | CATTGGGTGGCTTCATTCTC | CCTGCCTGACTTTAATACTTACCTT | 215 |
| 49 | ACCCTTTAATTCTTTGCTTTCGAAGGT | TGTTTCTATAATACTTAGGGCCATG | 249 |
| 50 | ACTGAGGACAGAAAAGTATCCCC | GTTCTGTCCTAGGCCGCTTC | 222 |
| 51 | TGAATACCACGCTTTATGAGAAGTT | AGTCAGAGCATACTACATAGATCCA | 244 |
| 52 | ACCTGCTGAGAAAACATGCC | ACTAATTGTACTCACGCCTTCCT | 245 |
| 53 | ATCAGAATGCCTGTTTGTGGT | CACTTAACCTGTACTATCAGGGTCA | 219 |
| 54 | ATGCATAACTCATTGTTGTCCTAGA | ACTGGCTGCTACAGGTGA | 246 |
| 55 | TAGCAGCCAGTCCCGTTT | ACATGTCTCCCAAATGTCACC | 249 |
| 56 | CAGCCTGCAAGTCAGTCCA | TTTACAATGTGCAATACATGTAGGGA | 244 |
| 57 | TAAGAAATAACAGAAAGCCTGGGTGAC | CGTGAGTAGGCAAGACATTTATTCACT | 187 |
| 58 | ATGTCTTGCCTACTCACGTC | AGCTTCAGATTCCGTAACTGGTG | 150 |

**Supplementary Table 2.** Variants in other PCG-related genes identified by whole exome sequencing.

| Family ID | Patient ID | Gene symbol | Chromosomal Position | Exons | DNA change | Amino acid change | Inheritance | gnomAD_all | gnomAD_eas | CADD | GERP++ |
| --- | --- | --- | --- | --- | --- | --- | --- | --- | --- | --- | --- |
| 11 | C-354 | CYP1B1 | Chr2: 38302213 | Exon2 | c.C319G | p.L107V | F | 0.00034 | 0.0045 | 23.5 | 2.73 |
| 10 | C-350 | CYP1B1 | Chr2: 38301574 | Exon2 | c.958G>T | p.V320L | F | 0.00029 | 0.0036 | 27.3 | 4 |
| 10 | C-350 | LTBP2 | Chr14: 74989495 | Exon16 | c.2657C>A | p.T886K | M | 0.00054 | 0.0029 | 21.3 | 2.88 |

Chromosomal positions correspond to GRCh37/hg19 assembly. Reference CYP1B1 mRNA sequence, NM_000104.4. Reference CYP1B1 protein sequence, NP_000095.2. Reference LTBP2 mRNA sequence, NM_000428.3. Reference LTBP2 protein sequence, NP_000419.1. gnomAD_all, allele frequency in all populations from gnomAD dataset. gnomAD_eas, allele frequency in East Asian populations from gnomAD dataset. CADD, Combined Annotation Dependent Depletion. GERP, The Genomic Evolutionary Rate Profiling. F, father. M, mother.

**Supplementary Table 3**. Summary of functional evaluation of identified TEK variants.

| Variants | Protein expression | solubility | Auto-phosphorylation | Response to ANGPT1 stimulation | Response to proteasomal inhibition | Pathogenicity |
| --- | --- | --- | --- | --- | --- | --- |
| p.H52R | - | - | - | + | / | - |
| p.M131I | - | - | - | + | / | - |
| p.M228V | - | - | - | + | / | - |
| p.P244S | ↓ | - | IF↑ | - | + | Lof |
| p.C264F | ↓ | - | IF↑ | - | + | Lof |
| p.H494Y | - | - | - | + | / | - |
| p.L504P | ↓ | - | IF↑ | - | - | Lof |
| p.A841V | / | / | / | / | / | Lof* |
| p.L888P | - | - | - | + | + | - |
| p.R1003H | ↑ | insoluble | SF↑ | + | / | Gof |
| p.Y1024* | / | / | / | / | / | Lof |

/, not tested; IF, insoluble fraction; SF, soluble fraction; Lof, loss-of-function; Gof, gain-of-function.

*This variant was first reported and studied by Young et al. (Young et al., 2020).
